# An Autonomous Localization Vest System Based on Advanced Adaptive PDR with Binocular Vision Assistance

**DOI:** 10.3390/mi16080890

**Published:** 2025-07-30

**Authors:** Tianqi Tian, Yanzhu Hu, Xinghao Zhao, Hui Zhao, Yingjian Wang, Zhen Liang

**Affiliations:** 1 Key Laboratory of IoT Monitoring and Early Warning, Ministry of Emergency Management, Beijing University of Posts and Telecommunications, Beijing 100876, China; tiantianqi@bupt.edu.cn (T.T.); wangyingjian@bupt.edu.cn (Y.W.); liangzhen@bupt.edu.cn (Z.L.); 2 School of Intelligent Engineering and Automation, Beijing University of Posts and Telecommunications, Beijing 100876, China; 3 China National Institute of Standardization, Beijing 100191, China; zhxhbupt@163.com; 4 Beijing Key Laboratory of High Dynamic Navigation Technology, Beijing Information Science and Technology University, Beijing 100192, China; zhhui@bistu.edu.cn

**Keywords:** autonomous localization, adaptive pedestrian dead reckoning, visual inertial odometry, binocular vision, step length estimation

## Abstract

Despite significant advancements in indoor navigation technology over recent decades, it still faces challenges due to excessive dependency on external infrastructure and unreliable positioning in complex environments. This paper proposes an autonomous localization system that integrates advanced adaptive pedestrian dead reckoning (APDR) and binocular vision, designed to provide a low-cost, high-reliability, and high-precision solution for rescuers. By analyzing the characteristics of measurement data from various body parts, the chest is identified as the optimal placement for sensors. A chest-mounted advanced APDR method based on dynamic step segmentation detection and adaptive step length estimation has been developed. Furthermore, step length features are innovatively integrated into the visual tracking algorithm to constrain errors. Visual data is fused with dead reckoning data through an extended Kalman filter (EKF), which notably enhances the reliability and accuracy of the positioning system. A wearable autonomous localization vest system was designed and tested in indoor corridors, underground parking lots, and tunnel environments. Results show that the system decreases the average positioning error by 45.14% and endpoint error by 38.6% when compared to visual–inertial odometry (VIO). This low-cost, wearable solution effectively meets the autonomous positioning needs of rescuers in disaster scenarios.

## 1. Introduction

As a critical position estimation technology, indoor navigation technology has been extensively applied in various fields such as disaster rescue, intelligent driving, industrial manufacturing, and aerospace in recent years [1,2]. By integrating data from multiple sensors, localization and navigation systems can accurately estimate the position and orientation of carriers, thereby providing reliable spatial positioning support for various application scenarios [3]. In disaster rescue operations, obtaining the position information of rescuers accurately and reliably not only significantly improves rescue efficiency but also reduces the risk of casualties in complex and dynamic disaster environments [4,5]. However, disaster environments are often characterized by complex spatial structures, severe infrastructure damage, and harsh environmental conditions, particularly in sheltered spaces, which exhibit “blind, narrow, chaotic, and dangerous” characteristics [6]. These extreme conditions hinder the effective application of existing localization and navigation technologies, failing to meet the critical need for autonomous localization and orientation of rescuers [7]. Consequently, research into autonomous localization and orientation for rescuers in disaster environments is paramount.

With the global expansion of urban underground space development, underground space disasters and accidents have become more frequent, posing severe threats to lives and property [8]. As leading countries in underground space development, China, the United States, and Russia face severe challenges from natural disasters and security incidents, with an urgent need for underground disaster rescue operations. Unlike surface rescue, disaster sites in urban underground spaces often exhibit “gray box” characteristics, where spatial structures undergo drastic changes and infrastructure collapses, rendering traditional rescue support methods, such as communication, lighting, and broadcasting, ineffective [9]. This results in extremely harsh rescue environments, increasing the difficulty of rescue efforts and significantly raising casualty risks [10]. Accident investigation reports indicate that, aside from unavoidable factors, rescuers in underground disaster spaces often experience limited cooperation due to unclear positions and statuses, which is a major cause of casualties [11]. Therefore, developing localization and orientation technology for rescuers in complex spatial disaster sites has become a central focus for both the government and academia.

To address the need for localization and orientation in such complex spatial environments, numerous scholars worldwide have conducted extensive research. Currently, localization and orientation methods rely on various technologies, including global navigation satellite systems (GNSS) [12], WIFI [13], cellular networks [14], communication base stations [15], computer vision [16], inertial navigation systems (INS) [17], ultra-wideband (UWB) [18], laser [19], Bluetooth [20], and wireless local area networks (WLAN), among others. For instance, Alaei Yan et al. [21] proposed a hybrid GPS-INS method that improves localization accuracy in signal-intermittent environments by integrating GPS data and inertial measurements. Lan et al. [22] employed the WIFI signal strength for indoor localization by constructing a WIFI fingerprint database, thereby enhancing the system’s robustness in complex indoor environments. Dong et al. [23] developed a UWB high-precision localization system that performed excellently in open environments but experienced significant accuracy degradation in cluttered underground environments. Visual simultaneous localization and mapping (SLAM) technology has also been widely used for real-time location tracking, as demonstrated by Campos’ method [24], which effectively captures environmental features, although it performs unstably under poor lighting or drastic environmental changes. Additionally, Huang et al. [25] integrated low-energy Bluetooth (BLE) with mobile devices to implement a portable indoor localization solution, though its applicability is limited in disaster-stricken underground areas with damaged infrastructure.

However, the aforementioned technologies often perform poorly in urban underground disaster scenarios. GNSS and cellular signals often fail in shielded underground spaces, and WIFI signals in disaster sites are unstable, prone to failure, and have limited coverage [19,26,27]. Technologies such as Bluetooth, UWB, and WLAN rely on pre-deployed infrastructure, but in emergency situations, these facilities may be severely damaged or cannot be rapidly deployed, making it difficult to meet practical demand [28,29]. Although laser sensors offer high-precision localization capabilities, they tend to be bulky, costly, and energy-intensive, making them inconvenient for rescuers to carry and use in complex rescue environments [30,31]. Therefore, infrastructure-based navigation methods are difficult to apply effectively in underground disaster environments, failing to meet the demands of high-efficiency and reliable localization for emergency rescue, underground tunnel inspections, and exploration of unknown areas.

In recent years, pedestrian autonomous localization systems that operate without relying on infrastructure have attracted considerable attention [32,33]. The primary challenge for such systems lies in maintaining high precision during long-distance tasks. As a result, visual navigation and inertial navigation have become central areas of focus within the field of autonomous navigation research. However, inertial navigation systems suffer from error accumulation over time, while visual navigation can experience localization divergence in environments with weak textures. To address these challenges, this paper proposes an autonomous navigation method tailored for complex spaces, leveraging APDR and visual localization. Specifically, the proposed method integrates advanced APDR and enhanced visual localization method to mitigate the issues of localization divergence in complex environments and error accumulation in inertial sensors. It exhibits strong adaptability and high localization accuracy, capable of meeting the localization needs of rescuers in complex environments. Our contributions are summarized as follows:

(1) To improve positioning accuracy, we collected multi-source sensor data from various body parts of rescue personnel under different motion states. A data feature discriminability model was constructed to analyze the differences among multiple characteristic parameters. Based on the analysis results, the optimal placement position for the data acquisition unit and the input feature parameters for the adaptive step length estimation model were selected.
(2) To address the limitations of traditional adaptive step length estimation methods specifically, poor adaptability to different motion patterns and low estimation accuracy, an improved adaptive step length estimation model is proposed. By analyzing data characteristics and applying multiple constraint conditions, the model achieves adaptive extraction of step length intervals. The step length estimation model was trained using selected input features with significant feature discriminability, enabling accurate estimation across various motion patterns and step lengths.
(3) This paper innovatively integrates step length features into the visual localization and tracking algorithm. Based on step length information, the fusion processing is implemented from three aspects: keyframe insertion mechanism, translation component constraint, and feature matching search region constraint. These strategies collectively ensure the accuracy and robustness of the visual tracking system in complex environments. Additionally, a wearable autonomous localization vest system is designed and tested in indoor corridors, underground parking lots, and tunnel environments.

The remainder of this paper is organized as follows: Section 2 introduces the overall framework of the proposed localization system. Section 3 details the data analysis and preprocessing. Section 4 introduces the methodology of our methods. Section 5 presents the experiments and results. Finally, the conclusion is given in Section 6.

## 2. System Overview

**Figure 1:**
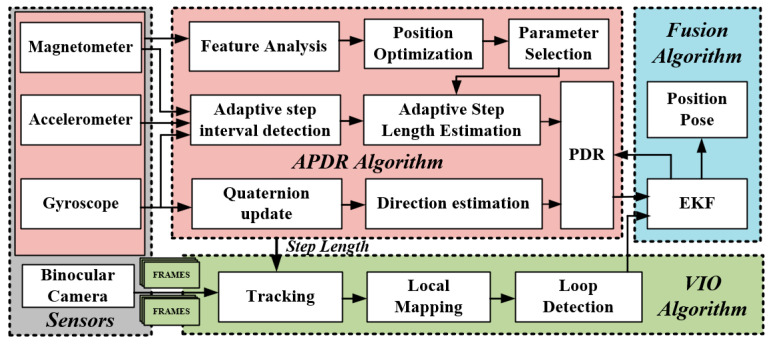
Framework of the proposed localization system.

This paper presents an autonomous localization system for rescuers, which enhances and integrates APDR and visual localization techniques. The overall framework of the system is illustrated in Figure 1 and consists of four primary modules, which are described in detail in the following sections.

The first is a sensor module composed of a magnetometer, accelerometer, gyroscope, and binocular camera. These sensors are mounted on the front chest area of the soldier’s vest to collect multi-source raw data during movement, including three-axis magnetic field strength, three-axis acceleration, three-axis angular velocity, and binocular image data.

The second is the advanced APDR module, designed to optimize the sensor layout and establish adaptive step length estimation and dead reckoning models. This module effectively acquires positional data by adaptively detecting step segmentation, optimizing sensor placement, adaptively estimating step lengths, and employing robust dead reckoning methods, significantly reducing the instability in localization accuracy that typically arises from step length estimation errors in conventional PDR systems.

The third is an enhanced VIO algorithm module that utilizes step length information to enhance the visual tracking process, aiming to improve the reliability and accuracy of position estimation. This module incorporates adaptive step length data into the visual positioning and tracking algorithm, specifically addressing the challenge of rapid divergence in traditional visual navigation systems, particularly complex environments. By increasing the stability and adaptability of the visual positioning module, it ensures reliable positioning even in challenging environments.

The fourth is the fusion module, which uses an EKF to integrate enhanced APDR with VIO. This integration achieves higher accuracy and more stable localization, significantly enhancing system performance in complex and occluded environments.

## 3. Analysis of Data Features

In this section, we analyzed the feature discriminability of multi-source data collected from different body parts of rescuers in various motion states. Based on the results of this analysis, we selected the optimal placement of data collection units and determined the appropriate feature parameters for the adaptive step length estimation model.

### 3.1. Analysis of Feature Discriminability

In rescue operations within confined or obstructed spaces, personnel engage in various activities such as crawling, crouch-walking, jumping, and running. The inertial data collected from different body parts during these movements exhibit significant variations in feature discriminability and extraction difficulty, directly impacting the accuracy of motion mode and step length recognition. To quantitatively analyze the feature discriminability across different motion states, multi-source data were collected, and a feature discriminability evaluation model was developed to assess the significance of key features under each motion state.

**Figure 2:**
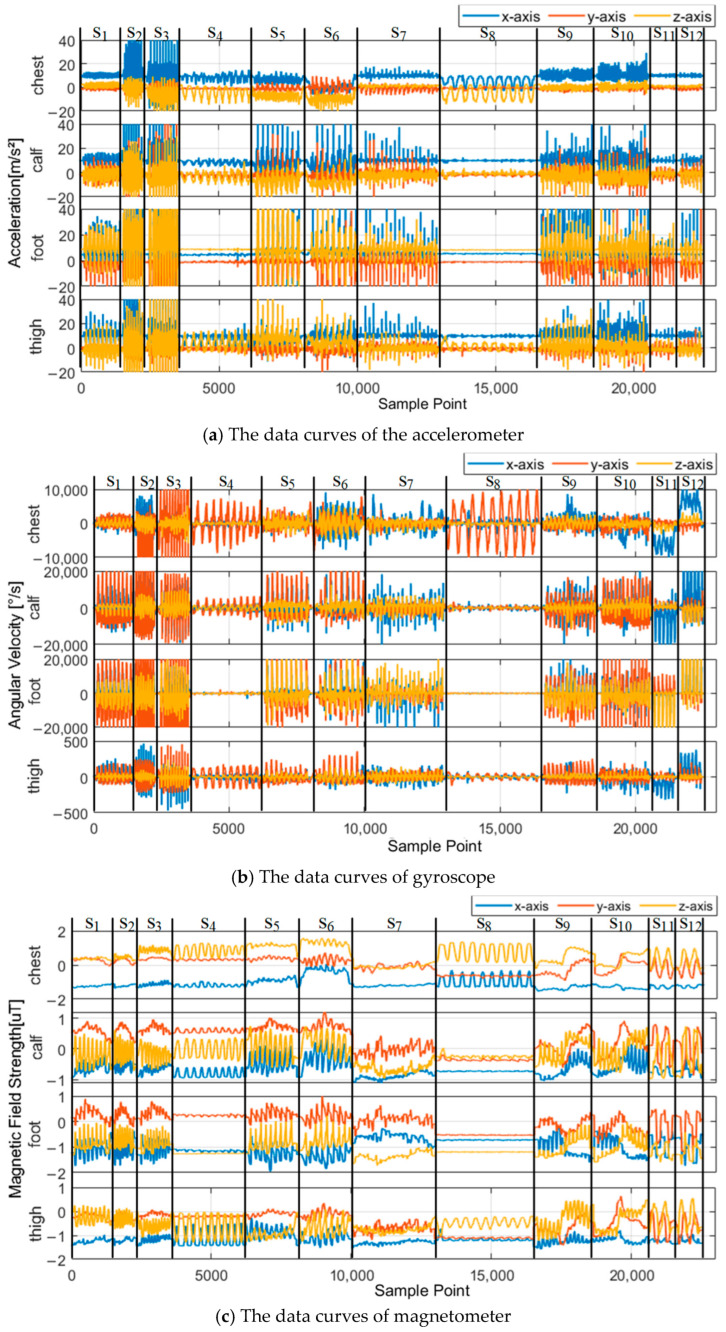
Inertial data curves of different body parts under various motion states.

To achieve this goal, multi-source sensors are installed on key body parts, including the chest, the outer side of the left thigh, the outer side of the left calf, and the top of the left foot. These sensors collect three-axis acceleration, three-axis angular velocity, and three-axis magnetometer data across a range of movements, including normal walking, running, jumping, squatting, moving while squatting, crawling, bending, side-stepping, going upstairs, going downstairs, turning in a circle to the right in place, turning in a circle to the left in place. The data of the motion states mentioned above are labeled as S_1_ to S_12_, respectively, as shown in Figure 2.

The feature discriminability model was constructed as follows:

Step 1: Calculation of Multi-Feature Parameters for Various Motion States

**Figure 3:**
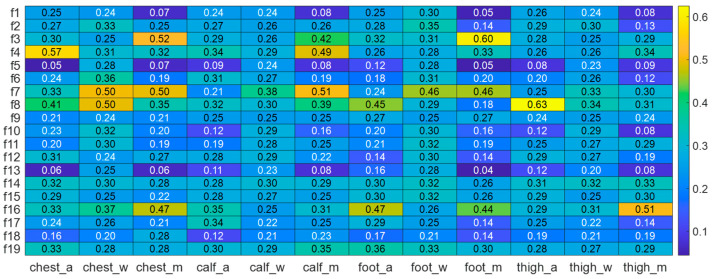
Feature discriminability heatmap of multi-source data collected from four body parts.

For each type of data, a set of characteristic parameters was computed, as outlined in Table 1. These features correspond to the identifiers f1–f19 in Figure 3.

Step 2: Standardization and Normalization

To ensure consistency in feature scaling, the extracted feature values were standardized and normalized:

1. Calculation of the mean feature value across multiple motion states:
  (1) $$\varepsilon =\frac{\sum_{i=1}^{n}\lambda_{i}}{n}$$
  where $\lambda_{i}$ represents the value of the *i*-th feature parameter, *n* is the number of states.

2. Standardization and normalization:
  (2) $$\mu_{i}=\frac{\lambda_{i}}{\varepsilon },\;\eta_{i}=\frac{\mu_{i}}{\max\{\mu_{1},\mu_{2},\ldots ,\mu_{n}\}},i=1,2,3,\ldots ,n$$

Step 3: Standard Deviation Calculation

To evaluate feature discriminability, the standard deviation was calculated as follows:

(3) $$\sigma_{s}=\sqrt{\frac{\sum_{i=1}^{n}{\left(\mu_{i}-\bar{\mu }\right)}^{2}}{n}}$$

Following the above procedure, the feature discriminability of each feature type from the three types of sensor data (acceleration, angular velocity, and magnetometer) was calculated across 12 motion states. Based on data collected from four body parts, a total of 228 feature discriminability values (19 features × 4 body parts × 3 sensor data types) were computed, as illustrated in Figure 3.

### 3.2. Optimization of Data Acquisition Unit Placement

The optimal placement of sensors is critical for acquiring data that is more conducive to stride length estimation across various motion states. Accordingly, we analyzed the feature discriminability across the four body locations based on the discriminability data presented in Figure 3. Specifically, we calculated the average differentiation values of acceleration, angular rate, and magnetometer features for each body part. Furthermore, we computed the overall average differentiation across all feature types for each body part, as summarized in Table 2. As shown in the table, the data collected from the chest exhibit more distinct characteristics compared to those from other body parts.

Furthermore, the data labeled as S_4_ and S_8_ in Figure 2 demonstrate more distinct features for squatting and bending. Specifically, the data collected from the chest demonstrate more pronounced and regular periodicity, with clearer and more comprehensive modal features. This characteristic helps improve the accuracy of motion state classification and aligns better with the practical needs of underground rescue scenarios. Additionally, chest data tend to be smoother, with lower noise levels and fewer interferences, facilitating the extraction of step data. In contrast, foot-mounted sensors are more susceptible to disruptions from complex post-disaster ground conditions, such as standing water, which can cause system malfunctions, and uneven terrain, which introduces instability in foot states and significantly reduces the signal-to-noise ratio.

During rescue missions, personnel often perform complex movements, such as bending, side-stepping, and crawling, when navigating confined spaces. These actions lead to more complicated motion states for the thighs and calves, reducing the reliability of these data for consistent motion classification. Considering these factors, this study identifies the chest as the optimal placement location for the autonomous localization and orientation data acquisition unit in rescue personnel.

### 3.3. Parameter Selection of Adaptive Model Dataset

An excessive number of features increases model complexity and may lead to overfitting, whereas too few features can result in underfitting. The primary goal of feature selection is to strike a balance by maintaining sufficient complexity for strong generalization performance while ensuring simplicity for efficient training, maintenance, and interpretation. When two or more features exhibit high correlation, multicollinearity arises, which can negatively impact model performance. Ideally, each feature in a machine learning model should be independent, minimizing or eliminating collinearity.

To optimize the training data structure of the adaptive step length estimation model and improve the adaptability and estimation accuracy of motion features and step length, this study quantitatively analyzes and selects the most relevant feature parameters based on data characteristics in Figure 3. This approach strikes a balance between data discriminability and computational efficiency.

To improve step length estimation, 12 key feature parameters with distinctive feature dissimilarity were selected from accelerometer, gyroscope, and magnetometer data from the device placed on the chest through data analysis. These features, chosen for their effectiveness in motion recognition and step length estimation, include six accelerometer-related parameters, three gyroscope features, and three magnetometer features. The selected features, which exhibit significant discriminability across 12 motion states, are as follows:

- Accelerometer features: The peak values of the data along the Y-axis and Z-axis, respectively; the trough values of the data along the Y-axis and Z-axis, respectively; skewness; and frequency band energy.
- Gyroscope features: The trough values of the data along the Y-axis and Z-axis, respectively; and skewness.
- Magnetometer features: The peak values of the data along the Y-axis; the trough values of the data along the Y-axis; and skewness.

Through this research, the optimal sensor placement and the most significant data types for input into the adaptive step length estimation model were identified. This approach enhances the model’s ability to estimate step length across various motion states, significantly reduces fluctuations in step length estimation errors, and improves both accuracy and robustness. Consequently, this method provides high-quality data support for accurately and reliably tracking personnel trajectories in subsequent applications.

## 4. Methodology

In this section, we present an autonomous positioning model that integrates adaptive pedestrian dead reckoning with vision-based localization. This method utilizes inertial data from rescuers to adapt to various motion states, with improvements in step detection and step length estimation. Furthermore, the model enhances visual localization techniques by incorporating step length information, thereby improving the accuracy and robustness of position estimation.

### 4.1. Adaptive Position Estimation

The adaptive position estimation for motion includes three primary components: step segmentation detection, adaptive step length estimation, and position estimation. First, based on the inertial data features collected from the chest of rescuers during movement, a step segmentation is defined. An adaptive step detection method is then proposed to accurately delineate step segmentations, adjusting to various motion states that may occur during the rescuer’s movement. Next, based on the results of the feature discriminability analysis and the selected twelve key features in Section 3, an adaptive step length estimation dataset is constructed. The parameters of the adaptive step length estimation model are then trained using this dataset. The improved step length estimation model is capable of adapting to multiple motion states such as walking, running, crouch-walking, and side-stepping. Finally, the estimated step lengths are integrated into a position estimation model to reconstruct the trajectory and estimate the real-time position of the individual.

#### 4.1.1. Step Segmentation Detection

In this study, step length is defined as the distance between the current foot position and the previous foot position when one foot of the pedestrian touches the ground. The corresponding step segmentation refers to the inertial data interval generated during that motion. The accuracy of step segmentation division directly affects the accuracy of step length estimation. To improve the accuracy of step segmentation, an adaptive peak detection method combining adaptive peak thresholding, adaptive time thresholding, and edge detection is designed to segment and extract step segmentation data.

(1) Adaptive Peak Threshold

**Figure 4:**
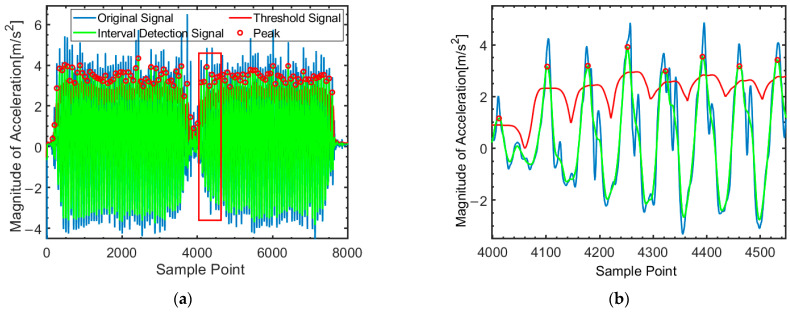
Adaptive peak threshold detection. (**a**) Comprehensive data curve overview. (**b**) Zoomed-in view of red-outlined area from left figure.

The accuracy of peak detection is primarily influenced by the pedestrian’s walking speed and the set threshold. This paper proposes an adaptive threshold calculation method based on the feature variations of chest inertial data, allowing the threshold to adjust according to changes in the pedestrian’s acceleration data. The adaptive threshold is set based on the standard deviation of the step detection signal within a sliding window and the maximum amplitude of the acceleration. This approach avoids the need to set separate thresholds for different motion states. The curve of adaptive threshold is shown as the red curve in Figure 4.

The standard deviation of the step detection signal is calculated as follows:

(4) $$\sigma_{s}(t)=\sqrt{\frac{1}{W}\sum_{t_{W}\in \Phi_{W}}{\left(\alpha_{s}(t_{W})-{\bar{\alpha }}_{s}(t)\right)}^{2}}$$

where $\sigma_{s}(t)$ represents the standard deviation of the step detection signal at the most recent sampling time *t*, *W* is the sliding window size, $\Phi_{W}(t)=\{t_{W}|t_{W}>-W\}$ represents the set of the latest *W* data sampling times, and ${\bar{\alpha }}_{s}(t)$ is the mean acceleration in the sliding window, calculated as:

(5) $${\bar{\alpha }}_{s}(t)=\frac{1}{W}\sum_{t_{W}\in \Phi_{W}(t)}\alpha_{s}(t_{W})$$

The filtered step detection signal’s peak is influenced by the walking speed. The faster speed, the greater standard deviation. The adaptive threshold formula is:

(6) $$\mu (t)=a_{s}\sigma_{s}(t)+b_{s}\alpha_{s}^{\max}+c_{s}\mu_{s}+d$$

where $\alpha_{s}^{\max}=\max\{\alpha_{s}(t_{W})\}$, $t_{W}\in \Phi_{W}(t)$ is the maximum value of acceleration amplitude in the sliding window, $\mu_{s}$ is the mean value of acceleration amplitude, and d is a constant to ensure that the adaptive threshold is no smaller than d. The coefficients $a_{s}$, $b_{s}$, $c_{s}$ and d were determined by fitting the model, and the final values of the parameters were set to 0.41, 0.2, 0.69, and 0.14, respectively.

(2) Adaptive Time Constraint

Since the pedestrian’s walking frequency remains within a narrow range, a reasonable time interval exists between adjacent peaks of the acceleration signal. However, sensor noise or irregular body movements may lead to the detection of multiple peaks within a single step period, while pauses or stops during walking can cause significantly longer intervals between consecutive peaks. A fixed time threshold may result in erroneous step segmentation. To address this issue, this paper proposes an adaptive time threshold method that dynamically adjusts the allowable peak interval based on the temporal characteristics of adjacent peaks, thereby reducing incorrect gait segmentation.

First, the time interval between the most recent adjacent peaks is calculated:

(7) $$t_{s}(i)=t_{s}^{p}(i)-t_{s}^{p}(i-1)$$

where $t_{s}(i)$ represents the time interval between the *i*-th and the previous peak of the step detection signal, and $t_{s}^{p}(i)$ represents the sampling time of the *i*-th peak in the acceleration signal.

The time interval $t_{s}(i)$ should satisfy the following constraint:

(8) $$(1-a_{t})\cdot T_{i-1}\leq t_{s}(i)\leq (1+a_{t})\cdot T_{i-1}$$

where $a_{t}$ is the constraint range parameter, and $T_{i-1}$ is the time threshold, updated as follows:

(9) $$T_{i}=\frac{T_{i-1}+t_{s}(i)}{2}$$

(10) $$a_{t}={\left(T_{i}-T_{i-1}\right)}^{2}$$

**Figure 5:**
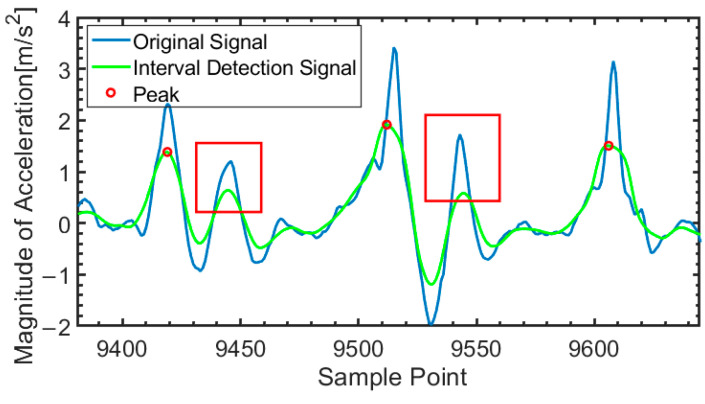
Adaptive time-constrained peak detection.

This time interval constraint effectively reduces the false detection rate of step segmentations. The results of the peak detection method with time constraints are shown in Figure 5. Peaks that do not satisfy the constraint conditions will be discarded, such as the peaks in the red frame.

(3) Edge Detection

Peak detection primarily relies on changes in the pedestrian’s center of mass. The improved peak detection method is capable of accurately identifying typical walking movements. However, when pedestrians perform unconventional actions, such as bending, squatting, or leaning backward, false peaks may be generated due to changes in their center of mass, which can lead to errors in step counting and position estimation. To mitigate such misdetections, edge detection based on the acceleration information before and after a peak is applied to minimize the impact of erroneous detections. A complete step segmentation should consist of the descent edge after the previous peak, the ascent edge, and the peak generated when the foot strikes the ground.

**Figure 6:**
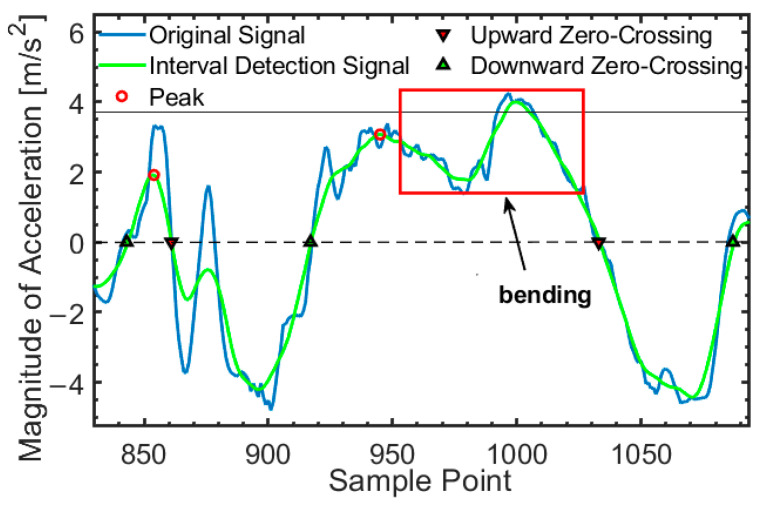
Edge detection constraints.

Figure 6 illustrates inertial data generated when a pedestrian bends during walking. The peak indicated by the arrow is caused by the sudden bending of the pedestrian. Since no descent or ascent edges were detected before this peak, it is discarded during edge detection, confirming that this data does not represent a valid step segmentation and should not be used for step length estimation or position calculation.

**Figure 7:**
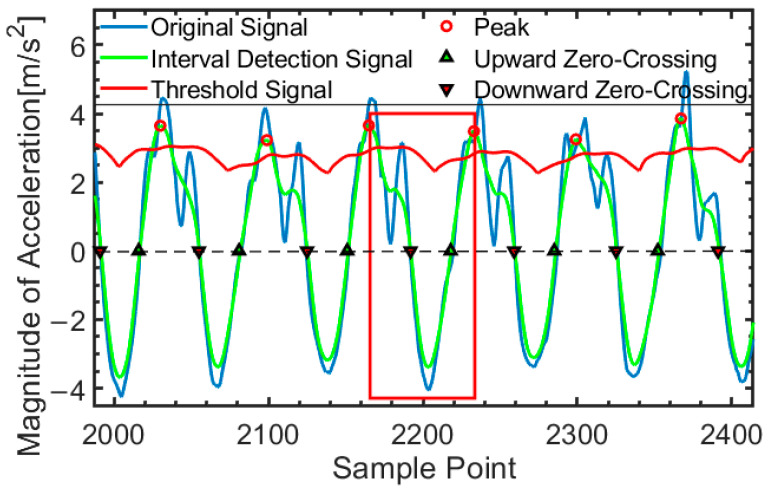
Step segmentation detection.

After filtering, the step detection signal shows a periodic sine wave, allowing step detection to be performed using the peaks. To reduce the false detection rate, multiple constraints are applied to accurately delineate the step segmentation, as shown in Figure 7. The step segmentation data is defined as the interval between two adjacent peaks. The conditions for a valid step segmentation include the presence of an upward zero-crossing, a downward zero-crossing, a time interval, and a peak caused by foot strike. The green curve segment within the red box represents the data of a single stride.

#### 4.1.2. Adaptive Step Length Estimation

The step length estimation model reflects the mapping relationship between the inertial data features within the step segmentation and the step length. This paper constructs a chest-mounted inertial dataset, extracts the corresponding data features within the step segmentation as input to a BP neural network, and trains the adaptive step length estimation model to output the estimated step length for the step segmentation.

In the experiment, test subjects wore IMU data acquisition units on their chests and performed various activities, including walking, jogging, jumping, crouch walking, crawling, side stepping, climbing stairs, descending stairs, bending, and turning left and right in place. During data collection, the step length of each step was calibrated. Since the IMU placement in existing pedestrian datasets does not match the setup used in this study, or the datasets involve a limited set of motion modes, a new dataset was constructed, and step lengths were calibrated. The calibration method involved placing markers on the inside of the subject’s ankles, leaving marks on the ground with each step, and measuring these marks to obtain the step length.

Using the step segmentation detection algorithm, the collected inertial data was segmented into step segmentations, and feature data was extracted to construct the training dataset for the adaptive step length estimation model. Twelve feature parameters from the step segmentations were selected for dataset construction. These feature parameters include Y and Z axis peaks from the accelerometer, Y and Z axis troughs from the accelerometer, accelerometer frequency band energy, Y and Z axis troughs from the gyroscope, Y axis peaks and troughs from the magnetometer, skewness from the magnetometer, skewness from the gyroscope, and skewness from the accelerometer.

**Figure 8:**
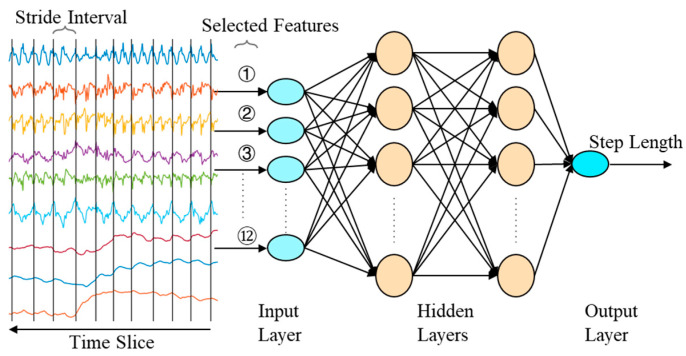
Diagram of the adaptive step length estimation model.

The BP neural network-based step length estimation model was constructed, as shown in Figure 8. The input layer contains 12 neurons, corresponding to the 12 input data features. The two hidden layers contain 20 and 10 neurons, respectively. The output layer contains 1 neuron, corresponding to the step length.

To evaluate the accuracy of the adaptive step length estimation model, the chest inertial data and step length measurements during the subjects’ movements were collected as a dataset. Specifically, we collected a total of 2520 steps of motion data, covering various locomotion modes such as walking, running, and crouching. For each step, the corresponding step length and segmentation points were labeled.

**Figure 9:**
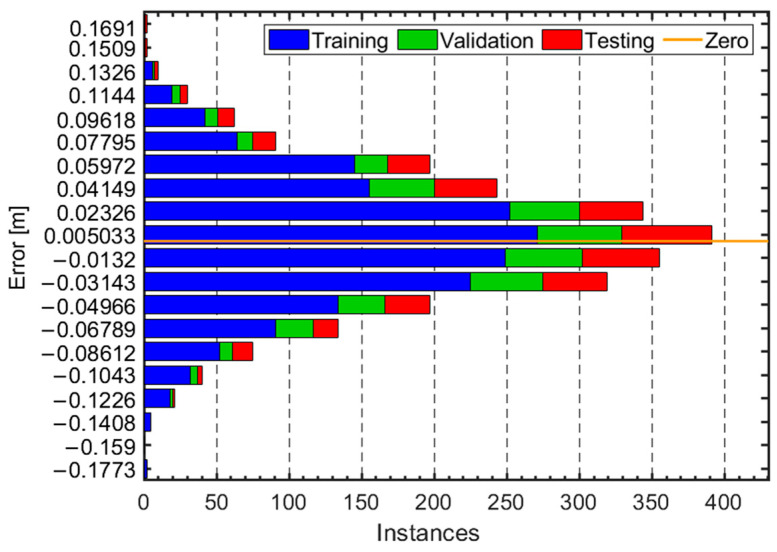
Histogram of errors during model training, validation, and testing.

To illustrate the model’s performance during the training, validation, and testing phases, we present Figure 9, which depicts the error distribution across different instances. Overall, the model demonstrates a fairly concentrated error distribution with most error values close to the zero-error baseline, highlighting high accuracy and robustness.

To further analyze the performance of our model, we calculated the Root Mean Square Error (RMSE) on the validation set. The computed RMSE is 0.047 m. This result indicates that our adaptive step length estimation model performs well in estimating pedestrian step lengths across various motion states. Such a low RMSE value suggests that the model is not only accurate but also robust in handling different types of human locomotion, including walking, running, and crouching.

#### 4.1.3. Position Estimation

**Figure 10:**
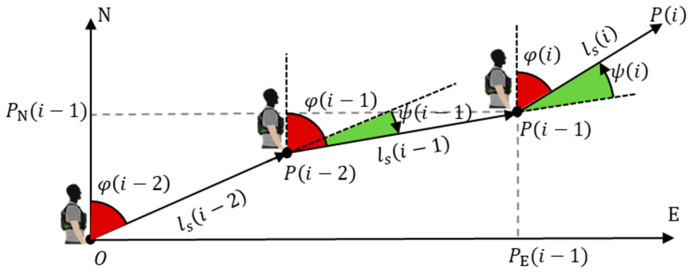
Diagram of the position estimation algorithm.

After completing step detection, step length estimation, and direction estimation, the pedestrian’s position can be calculated. Direction estimation is obtained through the processing of gyroscope angular rate data. Position estimation is performed by calculating the displacement relative to the previous step based on the pedestrian’s step length and direction, and then accumulating it with the previous position to update the current position. Figure 10 illustrates the position estimation algorithm.

The position estimation equations are as follows:

(11) $$P_{E}(i)=P_{E}(i-1)+l_{s}(i)\cdot \sin(\varphi (i))$$

(12) $$P_{N}(i)=P_{N}(i-1)+l_{s}(i)\cdot \cos(\varphi (i))$$

where φ(i)=φ(i−1)+ψ(i), ψ(i) represents the yaw angle, $P_{E}(i)$ and $P_{N}(i)$ represent the eastward and northward positions of the pedestrian for the *i*-th step, $l_{s}(i)$ represents the step length of the pedestrian for the *i*-th step, and φ(i) represents the direction of the pedestrian for the *i*-th step.

### 4.2. Vision-Based Localization Information Acquisition

In this section, we discuss visual localization technology constrained by step length. Existing vision localization techniques typically use data from cameras to generate localization trajectories through feature matching and tracking, local map construction, global map construction, and loop closure detection. In this paper, the improvements in the existing visual localization mainly focus on integrating step length into the tracking module and enhancing the feature match and keyframe decision of the module.

The primary task of the tracking module is to estimate the camera position in each frame and determine when to insert new keyframes. To fully utilize the step length of the rescuer and optimize the accuracy of camera position estimation, step length estimation is used to improve the accuracy and efficiency of feature matching, and refine the selection of keyframes. This method not only improves the accuracy of visual tracking for each step but also effectively prevents localization failure of the system due to large visual tracking errors occasionally. When the adaptive step length estimation model detects the completion of a step cycle, the visual localization system estimates the position of the camera within the current step cycle and applies step length to constrain the position.

**Figure 11:**
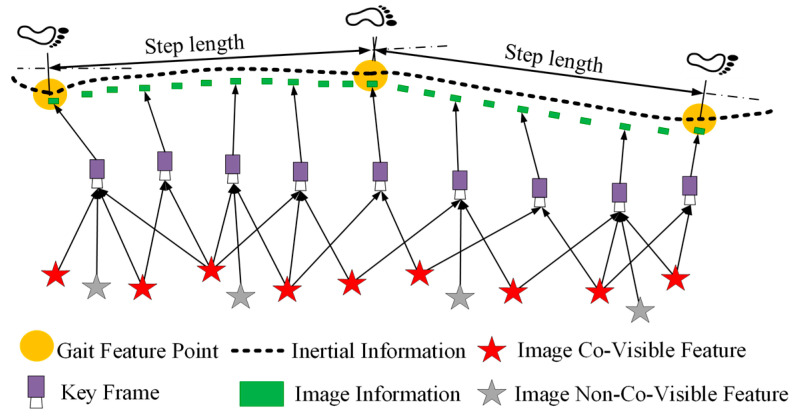
Diagram of vision-based position tracking principle with step length constraint.

In this process, the selection of keyframes is not only constrained by factors such as the number and distribution of feature matches, but also step length, aiming to improve the quality of keyframe extraction and the accuracy of feature matching. Figure 11 illustrates the principle of vision-based position tracking with step length constrain. This constraint mechanism of step length determines the selection of keyframes and image features, thereby improving the accuracy and stability of vision-based position tracking in complex environments.

By capitalizing on step length information, keyframe processing is conducted through three aspects: keyframe insertion mechanism, translation component constraint, and step-length-based feature matching search region constraint. These collectively guarantee the visual tracking system’s precision and robustness amidst complex scenarios.

#### 4.2.1. Keyframe Insertion Mechanism

A keyframe is forcibly inserted when the step length estimation model detects a complete gait cycle (e.g., peak in the gait segmentation signal, as shown in Figure 7), and the number of feature matches in the current visual frame $N_{f}$ is lower than a threshold $N_{T}$. This ensures that at least one visual keyframe corresponds to each walking step, maintaining map density. The threshold is dynamically adjusted based on the historical mean ($u_{f}$) and standard deviation ($\sigma_{f}$) of feature matching counts using the following formula:

(13) $$N_{T}=\max(u_{f}-k\cdot \sigma_{f},N_{T\_\min})$$

where *k* ∈ [1, 2] and $N_{T\_\min}$ = 15. When $N_{f}$ exceeds the threshold, keyframes are inserted conventionally; otherwise, keyframes are forcibly inserted. This method effectively increases the threshold in texture-rich scenes to reduce redundant keyframes, while lowering the threshold in texture-poor scenes (e.g., indoor corridors) to ensure environmental features are recorded.

#### 4.2.2. Step Length as a Visual Translation Component Constraint

Within each gait interval, the magnitude of the translation component $\Delta T_{\mathrm{vis}}$ in the visual pose transformation matrix should approximate the estimated step length $l_{s}$, that is, $||\Delta T_{\mathrm{vis}}|{|}^{2}\approx l_{s}$. If the error between them exceeds a threshold (e.g., $|||\Delta T_{\mathrm{vis}}|{|}^{2}-l_{s}|>0.2\;m$), the correction of the pose transformation matrix is triggered. The correction follows the formula:

(14) $$T_{c}=T_{\mathrm{vis}}\cdot \mathrm{Scale}(\frac{l_{s}}{||\Delta T_{\mathrm{vis}}|{|}^{2}})$$

where Scale(⋅) denotes the scaling function. This method effectively corrects visual drift caused by feature mismatches. For instance, if the actual step length is $l_{s}$ = 0.4 m, but the visual estimate is 0.7 m, the pose transformation is scaled down to approximately 0.4 m after incorporating the step length constraint, thereby correcting the drift.

#### 4.2.3. Step-Length-Based Feature Matching Search Region Constraint

During image feature matching within each gait interval, an adaptive step length estimation is used to constrain the search region for feature point pairs, improving both efficiency and accuracy compared to traditional ORB feature matching.

**Figure 12:**
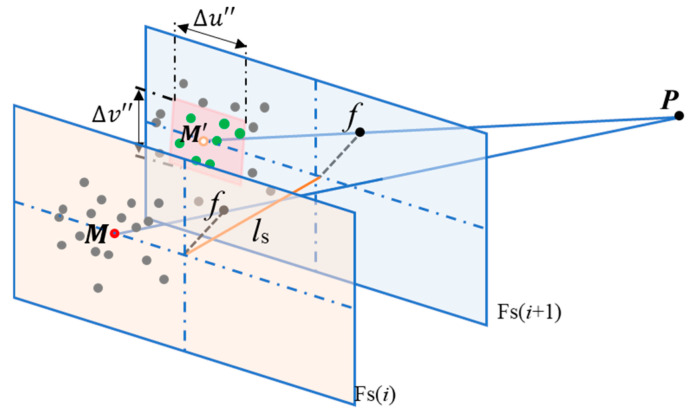
Schematic diagram of feature matching search region constrained by step length.

In this paper, the matched feature points are searched within a rectangular region centered on the predicted point. As shown in Figure 12, a feature point M(u,v) in the *i*-th keyframe Fs(i) will appear in the red rectangular region in the next keyframe Fs(i+1). During feature matching, only points within this region (green dots in Figure 12) are considered. f denotes the camera focal length; $l_{s}$ represents the step length; $\Delta u^{\prime \prime }$ and $\Delta v^{\prime \prime }$ are the width and height of the red rectangular region, respectively.

After one gait motion, the predicted pixel coordinates $M^{\prime }(u^{\prime },v^{\prime })$ of the corresponding feature point in the new frame Fs(i+1) can be estimated from the pixel coordinates M(u,v) in the old frame Fs(i), using the step length $l_{s}$, rotation matrix $R^{\prime }$, and camera parameters such as focal length f. Assuming a point $P(X_{w},Y_{w},Z_{w})$ in the world coordinate system has camera coordinates $P_{c}(X,Y,Z)$ with initial orientation matrix $R_{0}$ (pitch angle $\varphi_{0}$, yaw angle $\phi_{0}$, and roll angle $\theta_{0}$), and the camera moves along direction $d_{w}=(d_{x},d_{y},d_{z})$ by distance $l_{s}$ with rotation matrix $R^{\prime }$, then the coordinates ${P^{\prime }}_{c}$ of point $P_{w}$ in the camera coordinate system after the movement are:

(15) $${P^{\prime }}_{c}=(X^{\prime },Y^{\prime },Z^{\prime })=R^{\prime }(P_{c}-R_{0}\cdot d_{w}\cdot l_{s})$$

Taking forward motion as an example, where the heading aligns with the translation direction, the initial rotation matrix is identity I, and the movement direction is $\left(0,0,1\right)$. After moving $l_{s}$, the predicted pixel coordinates $M^{\prime }$ of feature point P' are given by:

(16) $$u^{\prime }=f\frac{X^{\prime }}{Z^{\prime }}=\frac{uZ}{Z-l_{s}}$$

(17) $$v^{\prime }=f\frac{Y^{\prime }}{Z^{\prime }}=\frac{vZ}{Z-l_{s}}$$

To validate the effectiveness of the search region constraint strategy described in the above, we evaluated the proposed Step-length-Based Feature Matching Search Region Constraint (SL-FSRC) algorithm using scenes containing repetitive structures or objects (e.g., chairs, ceiling corners, stairs, and markers), which are commonly found in real-world environments. In this approach, an appropriate search area is defined around the predicted pixel coordinates based on the step length. This limits the feature matching search and avoids exhaustive global searches typically used in traditional methods, thereby significantly reducing computational load. Furthermore, searching within a constrained region prevents erroneous matches caused by repetitive structures.

**Figure 13:**
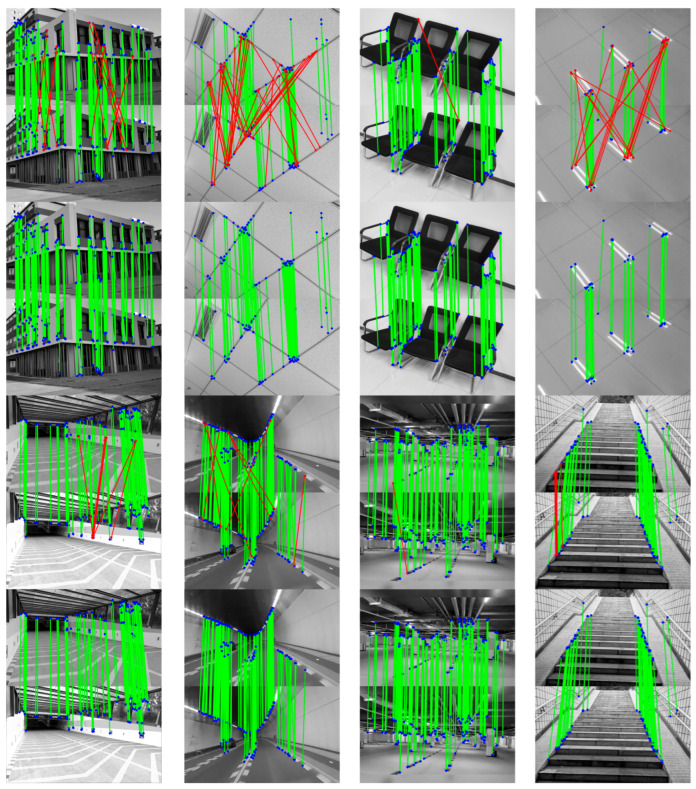
Visualization of feature matching results without step length constraint and with step length constraint in environments with repeating objects.

The traditional ORB algorithm was employed to detect feature points and perform feature matching based on Hamming distance. The matching results are illustrated in the first and third rows of Figure 13, where the red-dotted pairs indicate mismatches identified by the proposed constraint method when applied to the conventional matching results. The improved matching outcomes, obtained after removing these mismatches using the proposed SL-FSRC, are shown in the second and fourth rows of Figure 13. The results clearly demonstrate that the proposed algorithm effectively eliminates a large number of mismatched pairs caused by repetitive structures or cross-region ambiguities, thereby significantly improving the accuracy and reliability of the feature matching process.

### 4.3. Fusion

The EKF algorithm is employed for the fusion process. EKF is an enhancement of the traditional Kalman filter, with its core resembling the original Kalman filter, consisting of two main processes: time update and measurement update. Time update predicts the state variables and calculates the prior estimate of the covariance; the measurement update feeds back the estimation errors to the loop for state correction.

The state matrix is modeled using position δP (δPx,δPy,δPz), velocity δV (δVx,δVy,δVz), attitude δA (δθ,δφ,δψ), accelerometer bias errors δα ($\delta a_{x},\delta a_{y},\delta a_{z}$), and gyroscope bias errors δω ($\delta \omega_{x},\delta \omega_{y},\delta \omega_{z}$).

(18) $$X={\left[\begin{array}{ccccc} \delta P & \delta V & \delta A & \delta a & \delta \omega \end{array}\right]}^{T}$$

The system state equation is given by:

(19) $$X_{k+1}=FX_{k}+GW_{k}$$

(20) $$F=\left[\begin{array}{ccccc} I_{3\times 3} & \Delta t•I_{3\times 3} & 0 & 0 & 0\\ 0 & I_{3\times 3} & St•\Delta t & C_{b}^{n}•\Delta t & 0\\ 0 & 0 & I_{3\times 3} & 0 & -C_{b}^{n}•\Delta t\\ 0 & 0 & 0 & B_{1}•I_{3\times 3} & 0\\ 0 & 0 & 0 & 0 & B_{2}•I_{3\times 3} \end{array}\right]$$

(21) $$St=\left[\begin{array}{ccc} 0 & -a_{z} & a_{y}\\ a_{z} & 0 & -a_{x}\\ -a_{y} & a_{x} & 0 \end{array}\right]$$

where $X_{k+1}$ denotes the system state at time *k +* 1; F is the Jacobian matrix of the motion model with respect to the error and G is the Jacobian matrix with respect to the noise; $W_{k}$ represents zero-mean Gaussian white noise with covariance $Q_{k}$; $B_{1}$ and $B_{2}$ denote the measurement white noise of the accelerometer and gyroscope, respectively; I and 0 are the identity matrix and zero matrix, respectively.

The system measurement Equation is given by:

(22) $$Z_{k}=HX_{k}+V_{k}$$

(23) $$Z=\left[\begin{array}{c} P_{\mathrm{IMU}x}-P_{\mathrm{cam}x}\\ P_{\mathrm{IMU}y}-P_{\mathrm{cam}y}\\ P_{\mathrm{IMU}z}-P_{\mathrm{cam}z}\\ \theta_{\mathrm{IMU}}-\theta_{\mathrm{cam}}\\ \varphi_{\mathrm{IMU}}-\varphi_{\mathrm{cam}}\\ \phi_{\mathrm{IMU}}-\phi_{\mathrm{cam}} \end{array}\right]$$

where Z represents the measurement vector. $P_{\mathrm{IMU}x}$, $P_{\mathrm{IMU}y}$, $P_{\mathrm{IMU}z}$ denote the position information obtained by the inertial data; $P_{\mathrm{cam}x}$, $P_{\mathrm{cam}y}$, $P_{\mathrm{cam}z}$ denote the position information obtained by the camera data; $\theta_{\mathrm{IMU}}$, $\varphi_{\mathrm{IMU}}$, $\phi_{\mathrm{IMU}}$ are the roll, pitch, and yaw angles obtained by the inertial data, respectively; and lastly, $\theta_{\mathrm{cam}}$, $\varphi_{\mathrm{cam}}$, $\phi_{\mathrm{cam}}$ are the roll, pitch, and yaw angles obtained by the inertial device data, respectively. H denotes the measurement matrix; $V_{k+1}$ represents zero-mean Gaussian white noise with covariance $R_{k+1}$.

The matrix H can be expressed as

(24) $$H=\left[\begin{array}{ccc} I_{3\times 3} & 0_{3\times 6} & 0_{3\times 6}\\ 0_{3\times 3} & I_{3\times 3} & 0_{3\times 9} \end{array}\right]$$

The state matrix prediction Formula is:

(25) $$X_{k+1/k}=FX_{k}+GW_{k+1}$$

The measurement matrix prediction formula is:

(26) $$Z_{k+1/k}=HX_{k+1/k}+V_{k+1}$$

The covariance matrix prediction formula is:

(27) $$P_{k+1/k}=FP_{k}F^{T}+GQ_{k+1}G^{T}$$

where $Q_{k}=Q_{k}^{T}$ is the process noise covariance matrix

The Kalman gain matrix is computed as:

(28) $$K_{k+1}=P_{k+1/k}H^{T}{\left[HP_{k+1/k}H^{T}+R_{k+1}\right]}^{-1}$$

State estimation is updated as:

(29) $$X_{k+1}=X_{k+1/k}+K_{k+1}[Z_{k+1}-Z_{k+1/k}]$$

The covariance matrix is updated as:

(30) $$P_{k+1}=[I-K_{k+1}H]P_{k+1/k}{\left[I-K_{k+1}H\right]}^{T}+K_{k+1}R_{k+1}K_{k+1}^{T}$$

## 5. Experiments

In this section, we evaluate the effectiveness and positioning accuracy of the proposed rescuer localization system through experimental validation. The reliability and accuracy of the proposed algorithm are assessed by comparing its trajectory with those generated by the independent APDR and VIO algorithms. Experiments were conducted in a variety of environments, including indoor corridors, underground parking lots, and tunnels. This section also provides a detailed description of the experimental setup, procedures, results analysis, and discussion.

### 5.1. Experimental Setup

The vision/inertial module used in these experiments is the INDEMIND M1, developed by Beijing Indemind Technology Co., Ltd. based in Beijing, China. It offers advantages such as low cost and high data update rates, consisting of a tightly coupled binocular camera and IMU with microsecond-level synchronization precision. The binocular camera utilizes the OV9281 sensor from OmniVision Technologies, Inc. (located in Santa Clara, CA, USA), featuring a sampling frequency of 50 FPS and a resolution of 640 × 400. The IMU is ICM20602, model from TDK-InvenSense (formerly InvenSense Inc., based in San Jose, CA, USA), operating at a sampling frequency of 1000 Hz. Magnetic field data is collected using Xsens sensors from Xsens Technologies B.V. (situated in Enschede, The Netherlands) and transmitted wirelessly to the computing module.

**Figure 14:**
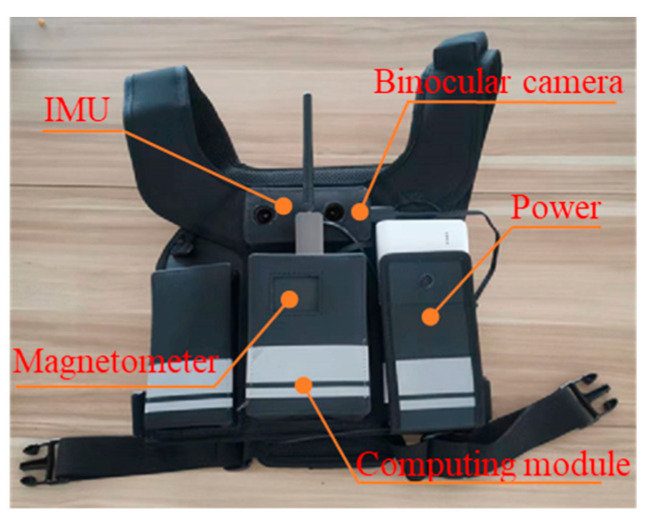
Device and module connections.

The computing module is an NVIDIA Jetson TX2 NX development board, produced by NVIDIA Corporation (headquartered in Santa Clara, CA, USA), which is installed with NVIDIA Jetpack 5.1 (also from NVIDIA Corporation), Ubuntu 20.04, and the ROS operating system. The INDEMIND M1 vision-inertial module is connected to the development board via a USB 3.0 interface, and the development board is powered by a 9 V power supply. The connections between modules are illustrated in Figure 14.

**Figure 15:**
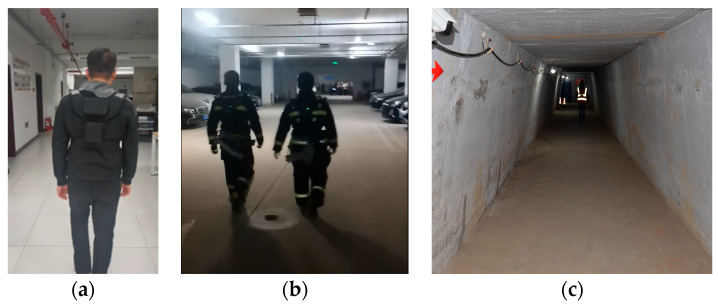
Testing environment. (**a**) Indoor corridors. (**b**) Underground parking lots. (**c**) Tunnels.

The device modules are securely attached via a custom wearable vest to reduce movement-induced device shake. The test personnel wear the integrated equipment, secure it on their chest, connect the power supply module, and turn on the power. After the computing module is powered on, the system automatically boots, starting the vision/inertial modules and the localization algorithm. The localization results are sent to the host computer via the wireless transmission module, and once the data is received, the test personnel begin their movement. The experimental scenario, in which personnel wore test vests to conduct trials, is shown in Figure 15a.

After completing the experiment, in order to facilitate comparison and error analysis, we calculate the average error, minimum error, maximum error, standard error, and endpoint error for each experiment. The verification tests were conducted in multiple scenarios, including indoor corridors, underground parking lots, and tunnels, as shown in Figure 15a,c.

### 5.2. Experimental Results

We employed three localization algorithms to estimate pedestrian positions: the APDR algorithm, the VIO algorithm, and the autonomous localization algorithm proposed in this paper. For the VIO algorithm, we utilized the latest ORB-SLAM3. The position error at each step was calculated, providing a detailed evaluation of localization accuracy.

Key evaluation metrics include average localization error, maximum error, minimum error, and standard deviation of error. These metrics allow for a comprehensive quantitative analysis of overall error levels, extreme error points, error fluctuation, and endpoint accuracy. They also facilitate a detailed comparison of the localization accuracy across different algorithms.

The experiments were conducted in multiple scenarios, which present diverse and complex settings. The presence of obstacles and dynamic environmental factors adds variability, thereby increasing the demand for algorithm reliability and stability. To evaluate the algorithm’s performance, three specific paths were chosen for testing: a straight-line round-trip, a curved path, and a closed-loop single circle. Each path posed unique challenges for the localization system. For instance, the straight-line round-trip path tested the consistency of straight-line motion and large-turn angle accuracy, validating the robustness of the algorithm in different typical motion states. The curved path assessed the algorithm’s localization accuracy in a dynamic, directional environment. Meanwhile, the closed-loop single-circle path was used to verify both the localization and orientation accuracy of the algorithm in a typical path.

**Figure 16:**
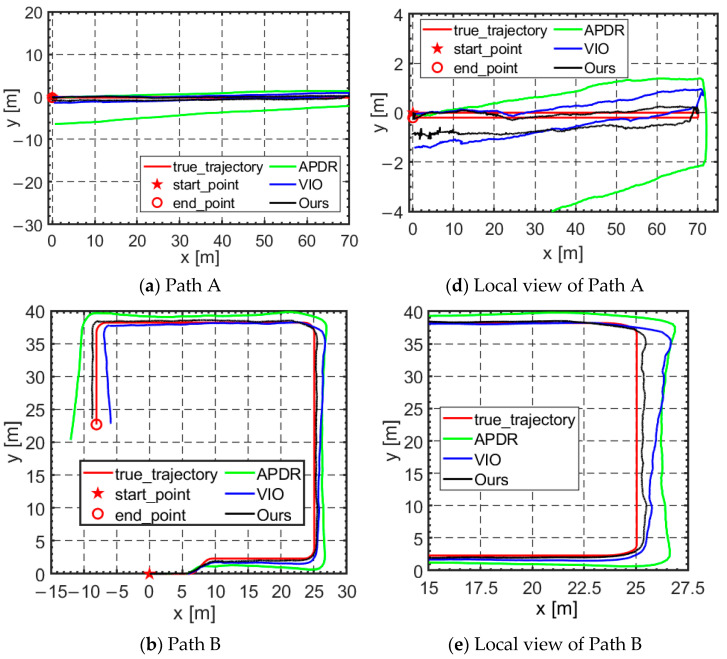

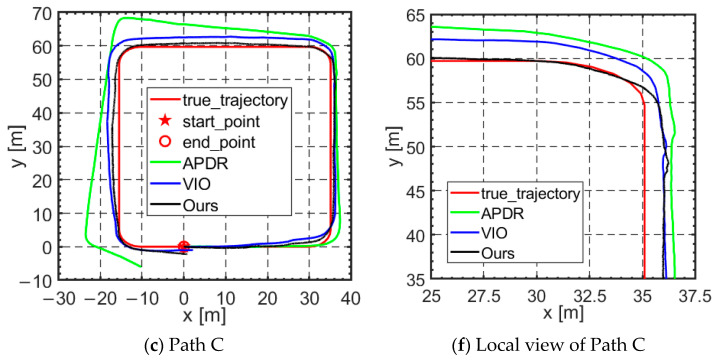
Trajectory curves for three different paths.

During the test, subjects wore the device and traveled along the three specified paths. The total distances for each path were as follows: straight-line round-trip 137.62 m, curved path 103.05 m, and closed-loop single circle 190.06 m. The trajectory results are shown in Figure 16a–c, with corresponding local views presented in Figure 16d–f. The trajectory diagram includes the true trajectory (red curve), the trajectory of the adaptive PDR algorithm (green curve), the trajectory of visual–inertial (blue curve), and the trajectory of our algorithm (black curve). The real trajectory was obtained by reconstructing the path using a foot-point marking tool and a laser ranging tool. Points were marked each time the heel hit the ground, with distances measured using the laser ranging tool to reconstruct the actual trajectory. 

By comparing the trajectories of three algorithms (APDR, VIO, and our proposed autonomous localization algorithm) with the real trajectory, the results demonstrate that the proposed fusion localization algorithm can track the real trajectory both stably and accurately. This highlights the algorithm’s consistency in tracking within complex environments, further validating its reliability and accuracy for real-world applications.

**Figure 17:**
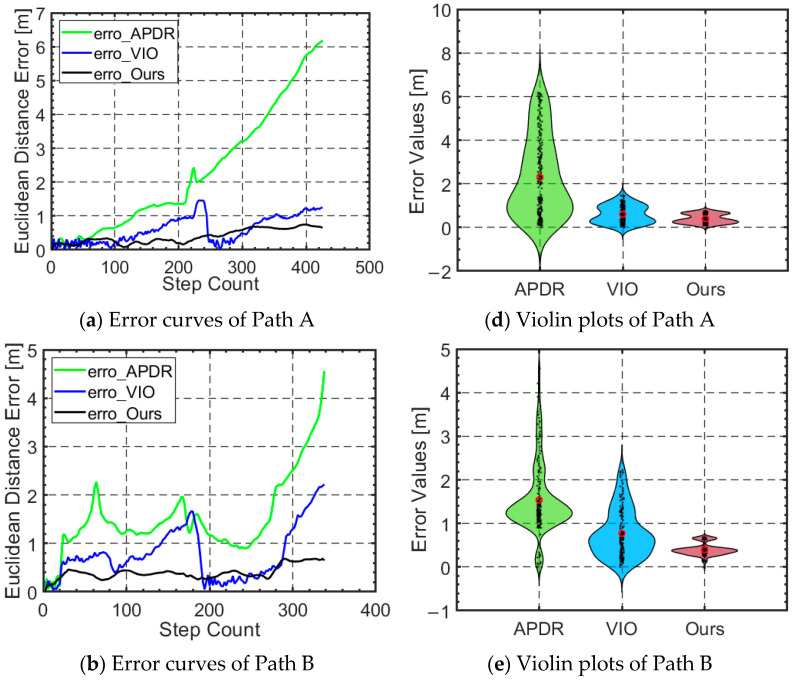

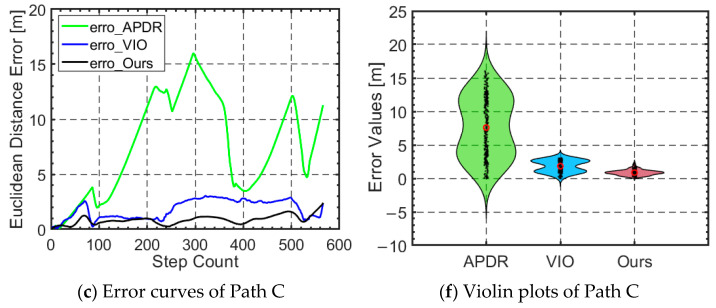
Localization error curves and violin plots for three different paths.

Figure 17a–f show the localization error over time for each trajectory, along with the corresponding error violin plots. From the error curves and distributions, it is evident that the fusion algorithm significantly reduces the error, and some of the error fluctuations exhibit more stability. Compared to the individual systems or algorithms, the fused algorithm achieves higher localization accuracy and stability, underscoring its greater potential for both practical applications and research value.

To quantify and compare the error levels, we performed a statistical analysis of the error data for three trajectories (APDR, VIO, and our proposed autonomous localization method) relative to the real trajectory. The analysis included maximum error, minimum error, average error, and error standard deviation. The error statistics are presented in Table 3.

The results in Table 3 clearly show that our proposed algorithm outperforms the individual VIO and APDR systems across all three paths. Specifically, in terms of overall error levels, the proposed algorithm achieved an average localization error of 0.56 m, which is significantly lower than the average errors of the APDR algorithm (3.82 m) and the VIO algorithm (1.07 m). The endpoint error was 1.24 m, which is substantially lower than the endpoint errors of the APDR algorithm (7.34 m) and the VIO algorithm (1.94 m). This demonstrates that the proposed algorithm offers better localization accuracy, with endpoint errors remaining within just 1.27%.

Regarding extreme error levels, our proposed algorithm achieved a maximum error of 1.28 m, significantly lower than the maximum errors of the APDR algorithm (8.91 m) and the VIO algorithm (2.24 m). This shows that the proposed algorithm performs better under extreme conditions, maintaining high accuracy even in complex or unfavorable environments, such as dynamic settings and rescue scenarios.

When considering error fluctuation, the proposed algorithm achieved a standard deviation of 0.24 m, which is significantly lower than the APDR algorithm (2.35 m) and the VIO algorithm (0.59 m). This indicates that the proposed algorithm offers more consistent and stable position estimates compared to the independent systems, demonstrating stronger stability and adaptability to changing external conditions.

Furthermore, the proposed algorithm not only outperforms the independent systems in terms of average localization error but also shows notable advantages over the state-of-the-art VIO algorithm. For instance, compared to VIO, our algorithm reduced the average error by 45.14% and the endpoint error by 38.6%, thereby achieving higher precision. This exceptional performance demonstrates that the system can effectively meet the autonomous localization needs of rescuers in complex spatial environments.

## 6. Conclusions

In this work, we proposed a novel autonomous personnel localization method to address the need for rescuers to achieve autonomous localization in complex environments. The proposed method integrates and enhances two technologies: PDR and VIO solutions. Additionally, we conducted feature selection for model training based on data characteristic analysis and established an adaptive step length estimation model to tackle the challenges of low accuracy and poor robustness in estimating rescuers’ step lengths in complex environments. Furthermore, step length data were incorporated into the visual localization algorithm to effectively improve the robustness of the visual localization system in complex environments. Subsequently, by employing a Kalman filter, we successfully fused VIO data with APDR data to achieve high-precision localization. Finally, a low-cost, wearable autonomous positioning vest system was designed, which demonstrated excellent performance during testing.

Our proposed solution facilitates autonomous localization of rescue personnel in complex post-disaster environments. Experimental results demonstrate that the proposed localization system significantly reduces localization errors and outperforms standalone PDR and VIO solutions in terms of overall error, extreme error, and error fluctuations. Moreover, compared to state-of-the-art VIO technologies, our system achieves superior localization accuracy.

Despite its effectiveness across multiple scenarios, the system still has certain limitations. Firstly, although our adaptive step length estimation and fusion algorithms have significantly reduced errors, challenges such as time synchronization issues and higher computational demands may arise in complex environments, potentially impacting real-time performance.

In future work, we aim to extend our method to more challenging environments, enhancing its adaptability and robustness in real-world complex scenarios. Furthermore, our ongoing efforts will focus on the integrated design of low-power and miniaturized sensors, developing an integrated MEMS sensor array that packages accelerometers, gyroscopes, magnetometers, and miniature binocular cameras on a flexible substrate to achieve lightweight and low-power goals for the vest system. Additional improvements may include integrating more sensor types, optimizing sensor fusion algorithms to boost localization accuracy and stability.

## Figures and Tables

**Table 1:** Characteristic parameters.

ID	Name	ID	Name
f1	peak value of amplitude (three-axis data)	f11	absolute mean (Y-axis)
f2	peak value (X-axis)	f12	absolute mean (Z-axis)
f3	peak value (Y-axis)	f13	mean of amplitude (three-axis data)
f4	peak value (Z-axis)	f14	variance of amplitude
f5	trough Value of amplitude	f15	standard deviation of amplitude
f6	trough value (X-axis)	f16	skewness of amplitude
f7	trough value (Y-axis)	f17	kurtosis of amplitude
f8	trough value (Z-axis)	f18	correlation coefficient between Y-axis and Z-axis data
f9	duration of step segmentation	f19	frequency band energy of amplitude
f10	absolute mean (X-axis)		

**Table 2:** The feature discriminability across the four body parts.

Types	Acc	Gyro	Mag	Mean
chest	0.2805	0.3105	0.2805	0.2905
calf	0.2505	0.2705	0.2805	0.2672
foot	0.2605	0.3005	0.2805	0.2805
thigh	0.2605	0.2605	0.2705	0.2638

**Table 3:** Trajectory error statistics.

Path	Method	Minimum Error (m)	Maximum Error (m)	Average Error (m)	Standard Deviation (m)	Endpoint Error (m)
Path A	APDR	0.08508	6.17711	2.30863	1.79395	6.17711
	VIO	0.02879	1.46039	0.60778	0.39585	1.24573
	OURS	0.07829	0.74569	0.39962	0.20471	0.66439
Path B	APDR	0.05205	4.55375	1.54539	0.78236	4.55375
	VIO	0.04364	2.21872	0.77853	0.53609	2.21872
	OURS	0.09929	0.68558	0.40072	0.13246	0.64760
Path C	APDR	0.07018	16.00896	7.61376	4.47056	11.27938
	VIO	0.05963	3.04747	1.82674	0.85140	2.36798
	OURS	0.13079	2.40783	0.86502	0.39598	2.40783

## Data Availability

The original contributions presented in the study are included in the article, further inquiries can be directed to the corresponding author.
